# Nutrient Patterns Associated with Fasting Glucose and Glycated Haemoglobin Levels in a Black South African Population

**DOI:** 10.3390/nu9010009

**Published:** 2017-01-19

**Authors:** Tinashe Chikowore, Pedro T. Pisa, Tertia van Zyl, Edith J. M. Feskens, Edelweiss Wentzel-Viljoen, Karin R. Conradie

**Affiliations:** 1 Centre for Excellence in Nutrition, North-West University, Potchefstroom 2520, North West Province, South Africa; tertia.vanzyl@nwu.ac.za (T.v.Z.); edelweiss-wentzel-viljoen@nwu.ac.za (E.W.-V.); karin.conradie@nwu.ac.za (K.R.C.); 2 Wits Reproductive Health and HIV Institute, University of the Witwatersrand, Johannesburg 2000, South Africa; ppisa@wrhi.ac.za; 3 Division of Human Nutrition, Wageningen University, P.O. Box 17, 6700 AA Wageningen, The Netherlands; edith.feskens@wur.nl; 4 Medical Research Council Research Unit for Hypertension and Cardiovascular Disease, Faculty of Health Sciences, North-West University, Potchefstroom 2520, South Africa

**Keywords:** plant based, fasting glucose and glycated haemoglobin, T2D, dietary patterns

## Abstract

Type 2 diabetes (T2D) burden is increasing globally. However, evidence regarding nutrient patterns associated with the biomarkers of T2D is limited. This study set out to determine the nutrient patterns associated with fasting glucose and glycated haemoglobin the biomarkers of T2D. Factor analysis was used to derive nutrient patterns of 2010 participants stratified by urban/rural status and gender. Principal Component Analysis (PCA) was applied to 25 nutrients, computed from the quantified food frequency questionnaires (QFFQ). Three nutrient patterns per stratum, which accounted for 73% of the variation of the selected nutrients, were identified. Multivariate linear regression models adjusted for age, BMI, smoking, physical activity, education attained, alcohol intake, seasonality and total energy intake were computed. Starch, dietary fibre and B vitamins driven nutrient pattern was significantly associated with fasting glucose (β = −0.236 (−0.458; −0.014); *p* = 0.037) and glycated haemoglobin levels (β = −0.175 (−0.303; −0.047); *p* = 0.007) in rural women. Thiamine, zinc and plant protein driven nutrient pattern was associated with significant reductions in glycated haemoglobin and fasting glucose ((β = −0.288 (−0.543; −0.033); *p* = 0.027) and (β = −0.382 (−0.752; −0.012); *p* = 0.043), respectively) in rural men. Our results indicate that plant driven nutrient patterns are associated with low fasting glucose and glycated haemoglobin levels.

## 1. Introduction

Type 2 diabetes (T2D) prevalence and the burden it places on populations is increasing globally, thereby making it a public health challenge which requires urgent attention [1]. For instance, the global prevalence of T2D among women increased from 5% to 7.9% from 1980 to 2014 [2]. The diabetes prevalence in Africa is projected to have the largest increase of 109% compared to other regions in the world by 2035 [3]. Urban black South Africans have not been spared from the T2D burden. The highest prevalence of T2D in Sub Saharan Africa was reported among urban black South Africans, with 60% of these cases being reported amongst women [4]. Black South African women also have the highest prevalence of obesity, which has been reported to be rising together with T2D in this population [5].

The adoption of Westernised lifestyles by urban dwellers is suggested to be among the leading factors resulting in the increase in non-communicable diseases (NCDs) such as T2D in developing countries [6]. The migration of populations from rural to urban areas has been accompanied by an increase in meat consumption as well as an increase in the consumption of sugary foods in South Africa [7]. Both of these foods are recognised as dietary risk factors for T2D [6]. However, improvements in micronutrient intake among black South African women in urban areas is suggested to be a result of the increased consumption of fruits and vegetables which are protective of T2D risk [8,9,10]. Thus, the role of diet as a risk factor for T2D is complex among the black South African women. In addition, people eat meals with a variety of nutrients which have interactive and synergistic effects on health [11]. Therefore, it is difficult to determine the separate effect of a food or nutrient on disease development as it is highly interrelated with other nutrients [11]. There is need of dietary pattern analysis methods which are able to evaluate the diet as a whole and clarify the effects of the consumption of sugary foods and meat products, together with improved intakes of fruits and vegetables, related to T2D risk amongst this population group.

Dietary pattern (or food pattern) approaches comprise data driven methods such as factor analysis, which allow the dietary information (or food intake) at hand to determine the unique dietary pattern for the population group being evaluated [10]. Although dietary patterns have been associated with disease risk, their effect is considered to be through nutrient intake; therefore, it is pivotal to determine nutrient patterns that are associated with T2D risk [12]. This information will aid in the understanding of the aetiology of T2D. The foods that people eat are governed by their cultural norms and beliefs, which vary amongst ethnic groups, making dietary patterns limited and not applicable across divergent population groups [12]. Evidence exists that the Western dietary pattern association with fasting glucose varies among different ethnic groups [13]. However, nutrients are universal, thereby making nutrient patterns associations with disease risk applicable for multiple population groups [14]. To the best of our knowledge, no study has reported on the association of the nutrient patterns with fasting glucose and glycated haemoglobin levels among apparently healthy individuals. T2D is a complex and multifactorial diseases, however glycated haemoglobin and fasting glucose levels are proxies for the development of this disease which are affected by diet [15]. Fasting glucose levels are indicative of the short term changes in glucose metabolism, while glycated haemoglobin depicts the long term changes [15]. This study seeks to evaluate the association of nutrient patterns derived by factor analysis with fasting glucose and glycated haemoglobin levels among apparently healthy black South Africans.

## 2. Materials and Methods 

### 2.1. Study Population

The study participants (*n* = 2010) were recruited from two urban and rural areas of the North West Province into the South African arm of the Prospective urban and rural epidemiological (PURE) study using a population based sampling strategy. Apparently healthy male and female volunteers between the ages of 35 and 60 years were recruited by the fieldworkers. Individuals were considered to be apparently healthy if they were not using any medication for chronic disease and if they were not diagnosed with a chronic medical condition/disease. The international PURE study is a large-scale epidemiological study, which comprises research participants recruited from 17 low, middle, and high income countries [16]. The South African arm of the PURE study was initiated in 2005 with initial five-year follow-up intervals up to 2015. At baseline in 2005 and at the five-year intervals during the course of the study, medical history, lifestyle behaviour (physical activity and dietary intake), blood collection (for both genetic and biochemical analyses), an electrocardiogram, and anthropometric assessments were performed to determine the role of risk factors in the development of cardiovascular diseases [16]. Our study was nested in the 2005 PURE study baseline data. Stratified nutrient pattern analysis was conducted according to gender and urban/rural status among the 2010 participants of the PURE study. However, significant associations of the nutrient patterns with fasting glucose and glycated haemoglobin were noted only among the rural women and rural men. Therefore, the detailed nutrient pattern and association results of the rural participants are elaborated in the main text while results of the urban men and women are illustrated in the Supplementary Materials.

### 2.2. Ethical Approval

The participants gave written informed consent before participating in the study and the study was conducted according to the Declaration of Helsinki principles [17]. Ethical approval was granted by the Ethics Committee of the North-West University, Potchefstroom Campus, with ethics number NWU-00016-10-A1.

### 2.3. Dietary, Anthropometric and Physical Activity Assessments

The trained fieldworkers captured the dietary intake of the participants using a standardised quantitative food frequency questionnaire (QFFQ) which had been validated for the same ethnic population group of the study participants [18,19]. The reproducibility of the QFFQ was also assessed and found to be good in this specific study population [20]. The dietary intake data were coded, analysed and nutrient intakes were computed using the South African Food Composition Database [21]. Body weight measurements were performed in duplicate by the PURE research team members using a portable electronic scale (Precision Health Scale, A & D Company, Tokyo, Japan), after which the mean was recorded. The heights of the subjects were determined by the PURE study research team members using a stadiometer (IP 1465, Invicta, and London, UK). The BMI of the participants was computed using the formula: BMI = weight (kg/height (m^2^)). The Baecke physical activity questionnaire (BPAQ) which was validated for South Africa was used to collect the physical activity information of the participants [22]. The questionnaire was used to compute a physical activity index score as described elsewhere [22,23]. This physical activity index scores were used in the multivariate regression analysis to adjust for physical activity.

### 2.4. Biochemical Measurements

The research participants were required to fast (at least 8 h with no food or beverages, including water before measurements) and their blood glucose levels were measured by the PURE research team. These fasting glucose levels were measured using the SYNCHRON^®^ System from fluoride plasma. The Bio-Rad D-10^TM^, HbA1c kit (Bio-Rad Laboratories, Inc., Hercules, France), which operates via cation exchange high performance liquid chromatography was used to assess HbA1c levels from whole blood ethylenediaminetetraacetic acid (EDTA) treated samples. The coefficient of variation of the glycated haemoglobin and fasting glucose tests were 1.16% and 2.1%, respectively.

### 2.5. Statistical Analysis

**Figure 1:**
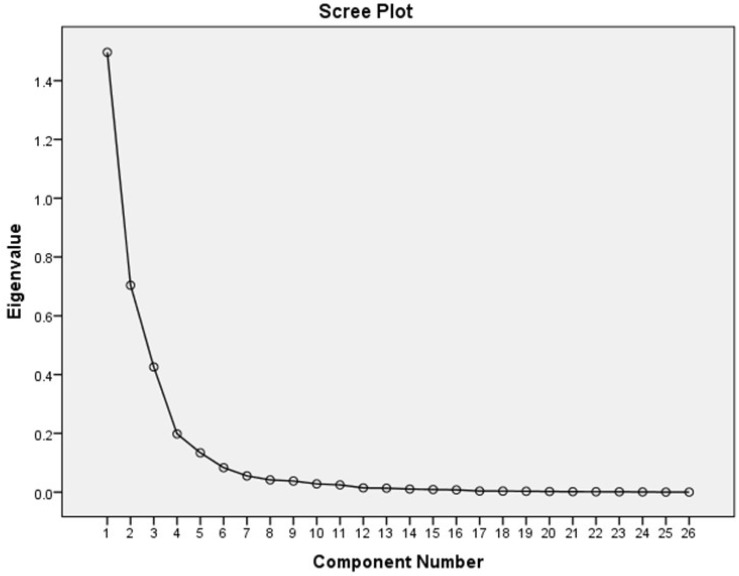
Scree plot of the nutrients and the extracted principal components among rural women.

The statistical analysis was performed using the statistical package for social scientists (SPSS) version 23. Normality tests for the continuous variables were performed using the Q-Q plots. Twenty-five nutrients were used to determine the nutrient patterns as has been reported previously by Pisa et al. [24]. Among these 25 nutrients, total protein was split into animal protein and plant protein; total carbohydrates were divided into total sugar, starch, and total dietary fibre; and total fat was categorised into saturated fat, monounsaturated fat and polyunsaturated fat. The total dietary fibre comprised soluble and insoluble dietary fibre. Alcohol was not regarded as a nutrient and not included in deriving the nutrient pattern analysis. However, in view of the reported association of alcohol intake with glycated haemoglobin, it was adjusted for in the multivariate linear models for the association of the derived nutrient patterns with glycated haemoglobin and fasting glucose [25]. The nutrient intake variables from the quantitative food frequency questionnaire (QFFQ) were log transformed to remove bias due to variance as a result of the different measures of scale used to quantify the nutrients. These nutrients were adjusted for log alcohol free energy using the multivariate (standard) method [11]. The multivariate method was selected as it yielded more cumulative variance and interpretable nutrient patterns compared to the standard nutrient density method of adjusting for total energy intake [11]. Principal component analysis (PCA) was the factor reduction tool used to determine the nutrient patterns from the 25 selected nutrients. The PCA was performed with the variance based on the covariance matrix and Varimax rotation. The retained principal components (PC) were used to identify the nutrient patterns. The scree plot (Figure 1) was used to determine the number of PCs to retain. The nutrient patterns were named using the nutrients with loadings greater than ±0.47 on the PCs. The total variances explained by the retained PCs were also evaluated to determine the relevance of the extracted PCs. The PCA was a suitable data reduction approach for the nutrient data in this study as was indicated by a Kaiser–Meyer–Olkin measure of sampling adequacy of 0.911, and a Bartlett’s test of sphericity which was significant at *p* < 0.001. The PCs were categorised into tertiles and analysis of variance ANOVA (for continuous variables) and Chi-square (for categorical variables) tests were used to determine the descriptive characteristics of the study participants across the tertiles of the extracted PCs.

Crude and adjusted multiple linear regression models were computed to assess the association between the extracted PCs with glycated haemoglobin and fasting glucose as dependent variables separately through varied models. In these models, regression coefficients for 1 standard deviation (SD) increase in the PC scores (and their 95% confidence intervals) were computed for four models: M1: (crude); M2: (adjusted for M1 plus Log Total Energy); M3: (adjusted for M2 plus Body Mass Index); and M4: (adjusted for M3 plus age, smoking, physical activity, education level, seasonality, alcohol intake and other PCs). Seasonality was adjusted based on the months in which the dietary assessments were conducted. Thus, for the rural participants, seasonality was adjusted basing on August and September; and the months of October and November were used to adjust for seasonality in the urban participants. Partial *R*^2^ values were computed to express the variance explained by each model. Statistical significance was regarded at *p* value less than 0.05.

## 3. Results

### 3.1. Nutrient Patterns

Three nutrient patterns (Table S1) were extracted from the PCA, which explained 73% of the total variation of the selected nutrient factors among the rural women. The first nutrient pattern was depicted as “Magnesium, phosphorus and plant protein driven nutrients”. This pattern consisted of higher loadings for magnesium, phosphorus and plant protein. The second nutrient pattern was termed “Fat and animal protein driven nutrients” as it had higher loadings of cholesterol, monounsaturated fat, animal protein, polyunsaturated fat and saturated fat. The “Starch, dietary fibre and B vitamins driven” based nutrient pattern was the third extracted nutrient pattern. This nutrient pattern had high loadings of starch, folate, vitamin B6, dietary fibre and thiamine as illustrated in Table S1.

Three nutrient patterns were extracted among rural men and named according to the nutrients with the highest loadings as indicated in Table S1. These were the “Thiamine, zinc and plant protein driven nutrients”, “Fat and animal protein driven nutrients”, and “Retinol and vitamin B12 driven nutrients”. Three similar nutrients patterns were extracted among the rural men, urban men and urban women, which explained 76%, 77% and 76% (Tables S1 and S2) variance of the nutrients, respectively. However, some considerable differences were noted in the plant driven nutrient patterns, which accounted for the greater variation of the nutrients among the urban and rural participants (Table S3).

### 3.2. Descriptive Characteristics of the Study Population

The study participants included 659 rural women, 347 rural men, 605 urban women and 399 urban men. The mean fasting glucose levels and glycated haemoglobin were 4.96 ± 1.57 mmol·L^−1^ and 5.64% ± 0.88%, respectively [26]. The women had a mean BMI of 26.73 ± 7.21, which signified that a considerable proportion of the women were overweight. Overall, in the whole study population, 39.4% of the participants were overweight and 87.3% of these were women. The mean total energy intake was 7707.13 ± 3692.36 KJ. The high scores of magnesium, phosphorus and plant protein driven nutrient pattern were associated with high total energy intake and energy from alcohol in rural women, as indicated in Table 1. The high scores for the fat and animal protein driven nutrient pattern was significantly associated with tertiary education status, as indicated in Table 1.

High scores for the thiamine, zinc and plant protein nutrient pattern were associated with higher energy and alcohol intake compared to other nutrient patterns among the rural men (Table 2).

### 3.3. Nutrient Patterns Associations with Fasting and Glycated Haemoglobin Levels

The association results of glycated haemoglobin and fasting glucose levels with 1 SD increases in the extracted nutrient patterns are shown in Table 3, Table 4, Table 5 and Table 6 for the rural women and rural men. The magnesium, phosphorus and plant protein driven nutrient pattern was associated with a consistent trend of increases in fasting glucose and glycated haemoglobin among rural women, as illustrated in Table 3.

Conversely, the starch, dietary fibre and B vitamins nutrient pattern was consistently associated with reduced glycated haemoglobin and fasting glucose levels in all evaluated linear models, for instance the M4 model: −0.175% (−0.303; −0.047); *p* = 0.007) and −0.236 mmol·L^−1^ (−0.458; −0.014); *p* = 0.037), respectively, as indicated in Table 3 and Table 4 among rural women.

The thiamine, zinc and plant protein nutrient pattern was associated with reduced glycated haemoglobin and fasting glucose levels in rural men as illustrated in Table 5 and Table 6. For instance, the M4 models, which explained the highest variances of the fasting glucose and glycated haemoglobin, indicated reductions of these outcome variables to be −0.382 mmol·L^−1^ (−0.752; −0.012; *p* = 0.043) and −0.288% (−0.543; −0.033; *p* = 0.027), respectively, as illustrated in Table 5 and Table 6.

The plant driven nutrient patterns had significant results as has been shown above among the urban and rural participants. However, some notable differences were depicted in the plant driven nutrient patterns illustrated in Table S3. The following nutrient patterns, “thiamine, zinc and plant protein driven nutrients”, “thiamine, starch and folate driven nutrients”, and “magnesium, phosphorus and plant protein driven nutrients” extracted from the urban men, urban women and rural women, respectively, had higher loadings for animal protein, saturated fat, mono-saturated fat and sugar compared to the “thiamine, zinc and plant protein driven nutrients” and “the starch, dietary fibre and B vitamin driven nutrients patterns”, which were associated with significant reductions in fasting and glycated haemoglobin among the rural men and women respectively (see factor loadings in bold in Table S3). These two nutrient patterns that had significant associations with fasting glucose and glycated haemoglobin had very low cholesterol and saturated fat as illustrated in Table S3.

## 4. Discussion

We set out to determine the nutrient patterns associated with fasting glucose and glycated haemoglobin levels among apparently healthy volunteers. The principal component analysis method enabled the extraction of three nutrient patterns among a black South African population which explained about 73% of the variation of the nutrient factors in the urban/rural and gender stratifications. The magnesium, phosphorus and plant protein driven nutrient pattern was associated with a trend of increasing fasting glucose and glycated haemoglobin levels per 1 SD increase in the pattern in the rural women while the thiamine, zinc and plant protein nutrient pattern was associated with a positive trend of increasing glycated haemoglobin among urban men. Notably, the starch, dietary fibre and B vitamin nutrient pattern was associated with decreases in glycated haemoglobin and fasting glucose levels, −0.175% ((−0.303; −0.047); *p* = 0.007) and −0.236 mmol·L^−1^ ((−0.458; −0.014); *p* = 0.037), respectively, among rural women. The thiamine, zinc and plant protein driven nutrient pattern was associated with significant reductions in fasting glucose and glycated haemoglobin of −0.382 mmol·L^−1^ (−0.752; −0.012; *p* = 0.043) and −0.288% (−0.543; −0.033; *p* = 0.027) in rural men. These associations were significantly maintained after adjusting for age, BMI, log total energy intake, smoking, physical activity, alcohol intake, seasonality and education level attained thus indicating an independent association of the starch, dietary fibre and B vitamin nutrient pattern and the thiamine, zinc and plant protein driven nutrient pattern with fasting glucose and glycated haemoglobin in the rural women and rural men strata’s, respectively.

Comparable studies evaluating the associations of nutrients patterns with fasting glucose and glycated haemoglobin are scarce. However, evidence exists of dietary patterns which have evaluated this phenomenon [10]. The Health/Prudent dietary pattern has been associated with decreases in fasting glucose and glycated haemoglobin levels while the Western dietary pattern has been associated with increases in these biomarkers of T2D [27,28]. However, the association of the Western dietary pattern, which is characterised with high intakes of animal proteins and snacks, with fasting glucose levels was noted to vary among ethnic groups [13]. The Western dietary pattern was reported in one study to be only significantly associated with high fasting glucose levels and glycated haemoglobin levels among Dutch and not among the Moroccans and Turkish groups [13]. Dietary patterns are known to vary among different ethnic groups, therefore nutrient patterns that were considered in our study are considered helpful in indicating a non-ethnic insight into this phenomenon.

In our study, we noted that fat and animal protein driven nutrients were also not associated with fasting glucose and glycated levels, as had been depicted in dietary pattern analysis studies among different ethnic groups [13]. This disparity from the Western populations where the animal based/Western dietary patterns are associated with increasing fasting glucose levels has been explained by the realisation that ethnic groups such as Asians may adopt the Western diets but their intake of animal products will remain lower as they also continue to consume traditional cereals and vegetables [29]. Similarly, in this study population, the intake of fat and animal protein nutrients were lower in the plant driven nutrients patterns that accounted for the greatest variance among the three nutrients patterns extracted per stratum as illustrated in Table S3. In other local studies, the protein and fat intakes as percentage of total energy intake for urban women from 1975 to 2005 did not change drastically though evidences of the adoption of Western dietary patterns were being noted and the greater proportion of the energy intake was still being contributed by the carbohydrate intake as had been noted in the Asians [6]. From 1975 to 2005, the percentage of energy of protein intake changed from 14% to 13%, while fat intake as percentage of energy changed from 21% to 30% and carbohydrate intake changed from 67% to 57% of total energy intake [6]. Thus, the intake of fat and animal protein driven nutrients in this population group might have been lower and thus not associated with increases in fasting glucose and glycated haemoglobin as was expected.

The magnesium, phosphorus and plant protein driven nutrient pattern indicated a positive trend association with increases in fasting glucose and glycated haemoglobin levels among rural women, while the thiamine, zinc and plant protein driven nutrient pattern was associated with a trend of increasing glycated haemoglobin levels in the urban men (Table S7). However, plant protein and zinc, which were high in these nutrient patterns, have been reported to independently lower fasting glucose levels [30,31,32,33]. In addition, the thiamine, zinc and plant protein driven nutrient pattern was associated with a trend of decreasing glycated haemoglobin and fasting glucose levels in the rural men. In view that the study population comprised people who consumed diets with both animal and plant based nutrients and not purely vegetarians, the discrepancies in the associations of the plant driven nutrients can be explained by the varied proportions of animal protein, saturated fat, mono-saturated fat, cholesterol and sugar among these plant driven nutrient patterns, as illustrated in Table S3. The magnesium, phosphorus and plant protein driven nutrient pattern in rural women and the thiamine, zinc and plant protein driven nutrient pattern in urban men had higher loadings of animal protein, saturated fat, mono-saturated fat, cholesterol and sugar compared to the other nutrient patterns discussed below such as the starch, dietary fibre and B vitamins driven nutrient pattern among rural women and the thiamine, zinc and plant protein nutrient pattern among rural men which associated with low fasting glucose and glycated haemoglobin levels. Animal protein, saturated fat, cholesterol and sugar are high in Western dietary patterns which have been previously associated with increased risk of T2D and this helps clarify the positive trend of association of the magnesium, phosphorus and plant protein driven nutrient pattern in rural women and the thiamine, zinc and plant protein driven nutrient pattern in urban men with the study outcome variables [34]. The comparisons of the varied constituents of the plant driven nutrients as discussed above and illustrated in Table S3 indicates the attractiveness of the PCA approach of nutrient pattern determination as it allows a whole based approach of the effect of nutrients to particular outcomes to be evaluated.

The starch, dietary fibre and B vitamins driven nutrient pattern was consistently associated with reduced fasting glucose and glycated haemoglobin levels in all the multivariate linear models which were considered in this study among rural women. The thiamine, zinc and plant protein nutrient pattern was also associated with significant reductions in glycated haemoglobin and fasting glucose among rural men. These findings are similar to the results of other studies on plant based dietary patterns and T2D risk [10,34]. It has been consistently reported that plant based diets are protective against T2D risk [10,34,35]. A number of mechanisms have been proposed to explain this phenomenon [10]. Plant based diets which are rich in dietary fibre are suggested to reduce T2D risk by reducing postprandial insulin demand, improving insulin sensitivity and the antioxidants found in these diets may help enhance β-cell function [36,37]. Dietary fibre from cereal foods which are also high in starch has been consistently shown across eight European countries to be associated with reduced T2D risk [38]. Maize meal fortified with iron, zinc and B vitamins which is largely consumed among the black South African population group is known to contain resistant starches that are partially digested and have been associated with improved insulin sensitivity which may then lead to reductions in fasting glucose levels [39,40]. Thiamine was also high in this nutrient pattern. Evidence exists that dietary fibre beneficial effects might also be due to its concomitant intake together with thiamine [41].The results of this study suggest that starch, dietary fibre and B vitamins nutrients, zinc and plant protein consumed together may lead to the lowering of fasting and glycated haemoglobin levels as has been postulated elsewhere [35].

The strengths of the current study include the use of a validated QFFQ and recruitment criteria of selecting apparently healthy individuals who were not taking chronic medications or suffering from chronic diseases. This might have helped to prevent the confounding of drugs and T2D to the nutrient pattern association results with glycated haemoglobin and fasting glucose levels. The factor analysis approach used to derive the nutrient patterns is known for depicting real-world dietary behaviours [42]. However, this approach is based on a number of subjective decisions such as naming nutrient patterns, method of rotation and selection of food groups which can lead to an overall measurement error [10,42]. Since this approach is a posteriori analysis tool, it derives nutrient patterns based on data at hand and this makes comparisons with other studies difficult [10,42]. However, regardless of the differences in the constituents of the nutrient patterns due to the data driven approaches used to derive them, consistent similarities of the dietary factors associated with T2D risk as reported in this study, has been depicted in multiple populations [10,34,37]. It might be probable that the residual confounding effect of total energy intake might have led to distortions in the association of the nutrient patterns with fasting glucose and glycated haemoglobin levels. Residual confounding is a result of measurement error in a confounder included in the model [43]. Although the nutrient and total energy intake based on the QFFQs are not precisely measured, adjustment for total energy intake should control for total energy intake confounding [44]. However, evidence exists that the control of total energy intake is not complete in epidemiological studies involving QFFQs thereby leading to residual confounding [44,45]. Therefore, there is need to further explore the association of the nutrient patterns in isocaloric clinical trials which are better designed to control for confounding for total energy intake [45].

## 5. Conclusions

In summary, our results indicate the beneficial associations of plant driven nutrient patterns with reductions in fasting glucose and glycated haemoglobin levels. However, the small variances that were explained by the models explored in this study are suggestive of the presence of other factors that affect the variation of fasting glucose and glycated haemoglobin which were not accounted for in this study. Thus, more studies are required to further explore the association of nutrient patterns with fasting glucose and glycated haemoglobin.

## Figures and Tables

**Table 1:** Descriptive characteristics for the rural women of the study population according to the lowest and highest tertiles of the three nutrient patterns (PC).

	Magnesium, Phosphorus and Plant Protein Driven Nutrients			Fat and Animal Protein Driven Nutrients			Starch, Dietary Fibre and B Vitamin Driven Nutrients		
	T1	T3	*p*	T1	T3	*p*	T1	T3	*p*
Age	48.72 ± 10.23	47.51 ± 9.57	0.247	47.94 ± 8.89	47.73 ± 10.16	0.837	49.15 ± 10.40	46.94 ± 8.99	0.036
Body Mass Index	25.28 ± 6.63	25.27 ± 6.58	0.988	25.31 ± 6.75	26.13 ± 6.85	0.262	25.26 ± 6.41	25.65 ± 7.11	0.605
Total energy	4322.96 ± 1217.30	8365.01 ± 2476.92	<0.001	5631.33 ± 2816.62	6883.88 ± 2189.38	<0.001	5893.08 ± 2645.21	6567.51 ± 2425.60	0.013
Alcohol (%TE)	0.76 ± 2.56	7.42 ± 12.56	<0.001	5.54 ± 11.70	1.47 ± 4.40	<0.001	5.36 ± 11.91	1.69 ± 5.01	<0.001
Protein (%TE)	11.26 ± 1.96	10.77 ± 1.64	0.008	10.27 ± 1.33	11.67 ± 1.81	<0.001	11.23 ± 2.05	10.91 ± 1.31	0.092
Current Smokers (%)	33.5	36.8	0.168	38.0	34.7	0.005	30.6	36.4	0.595
Physical Activity Index	8.19 ± 1.42	8.28 ± 1.42	0.593	8.29 ± 1.29	8.32 ± 1.55	0.854	8.23 ± 1.37	8.44 ± 1.45	0.205
Tertiary education (%)	33.7	24.5	0.058	26.5	42.9	0.028	28.6	38.8	0.854
Fasting glucose (mmol·L^−1^)	4.73 ± 0.78	4.93 ± 1.47	0.219	4.91 ± 1.18	4.87 ± 1.02	0.784	5.12 ± 2.41	4.88 ± 0.83	0.160
HbA1C (%)	5.64 ± 0.53	5.67 ± 0.93	0.785	5.68 ± 0.91	5.69 ± 0.58	0.843	5.84 ± 1.23	5.62 ± 0.55	0.027

*p* = *p* value based on ANOVA or Chi-square test where appropriate; Alcohol (%TE) = percentage of total energy due to alcohol intake; Protein (%TE) = percentage of total energy due to protein intake; T1 = lowest tertile; T3 = highest tertile, TE = total energy; HbA1C = glycated haemoglobin.

**Table 2:** Descriptive characteristics for the rural men of the study population according to the lowest and highest tertiles of the three nutrient patterns (PC).

	Thiamine, Zinc and Plant Protein Driven Nutrients			Fat and Animal Protein Driven Nutrients			Retinol and Vitamin B12 Driven Nutrients		
	T1	T3	*p*	T1	T3	*p*	T1	T3	*p*
Age	47.95 ± 9.99	50.19 ± 10.69	0.156	48.43 ± 9.97	51.42 ± 11.45	0.057	49.66 ± 10.01	49.57 ± 9.95	0.954
Body Mass Index	20.86 ± 4.15	20.54 ± 4.31	0.606	20.86 ± 3.99	20.94 ± 4.65	0.894	20.30 ± 3.57	20.95 ± 4.19	0.297
Total energy	4693.60 ± 1584.26	10,637.00 ± 2887.76	<0.001	6319.63 ± 3228.51	8220.85 ± 3228.51	<0.001	7164.14 ± 2975.31	7855.83 ± 3672.65	0.159
Alcohol (%TE)	5.98 ± 9.40	13.82 ± 13.70	<0.001	11.19 ± 13.41	5.70 ± 9.16	0.002	6.65 ± 11.23	9.19 ± 11.79	0.159
Protein (%TE)	11.51 ± 2.69	10.76 ± 1.53	0.014	10.09 ± 1.43	12.19 ± 2.33	<0.001	10.64 ± 1.69	11.50 ± 2.48	0.005
Current Smokers (%)	32.4	35.9	0.636	40.0	26.2	0.003	31.7	31.7	0.577
Physical Activity Index	8.25 ± 1.64	8.02 ± 1.44	0.380	8.03 ± 1.65	7.99 ± 1.79	0.861	7.92 ± 1.61	7.97 ± 1.63	0.847
Tertiary education (%)	44.2	20.9	0.141	23.3	37.2	0.514	27.9	37.2	0.451
Fasting glucose (mmol·L^−1^)	4.90 ± 0.93	4.68 ± 0.90	0.113	4.75 ± 0.79	4.96 ± 1.24	0.138	4.94 ± 2.41	4.75 ± 0.78	0.190
HbA1C (%)	5.59 ± 0.52	5.51 ± 0.81	0.386	5.49 ± 0.34	5.62 ± 0.97	0.169	5.57 ± 0.83	5.53 ± 0.49	0.678

*p* = *p* value based on ANOVA or Chi-square test where appropriate; Alcohol (%TE) = percentage of total energy due to alcohol intake; Protein (%TE) = percentage of total energy due to protein intake; T1 = lowest tertile; T3 = highest tertile; TE = total energy; HbA1C = glycated haemoglobin.

**Table 3:** Regression coefficients for fasting glucose for 1 SD increase in the derived nutrient pattern scores among rural black South African women.

	Magnesium, Phosphorus and Plant Protein Driven Nutrients			Fat and Animal Protein Driven Nutrients			Starch, Dietary Fibre and B Vitamin Driven Nutrients		
	B (95% CI)	*p* Value	*R*^2^	B (95% CI)	*p* Value	*R*^2^	B (95% CI)	*p* Value	*R*^2^
M1	0.129 (−0.014; 0.271)	0.077	0.007	0.009 (−0.141; 0.160)	0.902	0.000	−0.164 (−0.311; −0.018)	0.027	0.008
M2	0.196 (−0.063; 0.455)	0.138	0.007	−0.020 (−0.183; 0.143)	0.813	0.003	−0.197 (−0.349; −0.049)	0.011	0.016
M3	0.278 (−0.001; 0.280)	0.034	0.045	−0.038 (−0.198; 0.123)	0.645	0.037	−0.203 (−0.351; −0.054)	0.008	0.051
M4	0.147 (−0.360; 0.655)	0.569	0.086	−0.004 (−0.290; 0.281)	0.976	0.086	−0.236 (−0.458; −0.014)	0.037	0.086

M1: (crude); M2: (adjusted for M1 plus Log Total Energy); M3: (adjusted for M2 plus Body Mass Index); M4: (adjusted for M3 plus age, smoking, physical activity, alcohol intake, seasonality, education level, PC1, PC2 and PC3); M1 = model 1; M2 = model 2; M3 = model 3; M4 = model 4; PC1 = Magnesium, phosphorus and plant protein driven nutrients; PC2 = Fat and animal protein driven nutrients; PC3 = Starch, dietary fibre and B vitamin driven nutrients Fasting glucose units mmol·L^−1^ = millimoles per litre; SD = standard deviation; CI = confidence interval.

**Table 4:** Regression coefficients for glycated haemoglobin for 1 SD increase in the derived nutrient pattern scores among rural black South African women.

	Magnesium, Phosphorus and Plant Protein Driven Nutrients			Fat and Animal Protein Driven Nutrients			Starch, Dietary Fibre and B Vitamin Driven Nutrients		
	B (95% CI)	*p* Value	*R*^2^	B (95% CI)	*p* Value	*R*^2^	B (95% CI)	*p* Value	*R*^2^
M1	0.029 (−0.055; 0.112)	0.502	0.001	0.032 (−0.056; 0.120)	0.477	0.001	−0.138 (−0.224; −0.053)	0.002	0.020
M2	0.048 (−0.104; 0.199)	0.538	0.001	0.028 (−0.067; 0.123)	0.563	0.001	−0.145 (−0.234; −0.056)	0.001	0.021
M3	0.112 (−0.036; 0.260)	0.139	0.069	0.014 (−0.078; 0.106)	0.766	0.065	−0.151 (−0.237; −0.065)	0.001	0.087
M4	0.107 (−0.188; 0.401)	0.478	0.150	−0.011 (−0.175; 0.154)	0.478	0.150	−0.175 (−0.303; −0.047)	0.007	0.150

M1: (crude); M2: (adjusted for M1 plus Log Total Energy); M3: (adjusted for M2 plus Body Mass Index); M4: (adjusted for M3 plus age, smoking, physical activity, alcohol intake, seasonality, education level, PC1, PC2 and PC3); M1 = model 1; M2 = model 2; M3 = model 3; M4 = model 4; PC1 = Magnesium, phosphorus and plant protein driven nutrients; PC2 = Fat and animal protein driven nutrients; PC3 = Starch, dietary fibre and B vitamin driven nutrients. Glycated haemoglobin unit = per cent; SD = standard deviation; CI = confidence interval.

**Table 5:** Regression coefficients for fasting glucose for 1 SD increase in the derived nutrient pattern scores among rural black South African men.

	Thiamine, Zinc and Plant Protein Driven Nutrients			Fat and Animal Protein Driven Nutrients			Retinol and Vitamin B12 Driven Nutrients		
	B (95% CI)	*p* Value	*R*^2^	B (95% CI)	*p* Value	*R*^2^	B (95% CI)	*p* Value	*R*^2^
M1	−0.057 (−0.172; 0.057)	0.326	0.004	0.054 (−0.061; 0.169)	0.355	0.003	−0.039 (−0.156; 0.077)	0.504	0.002
M2	−0.237 (−0.492; 0.019)	0.069	0.013	0.061 (−0.064; 0.186)	0.335	0.004	−0.055 (−0.173; 0.064)	0.363	0.003
M3	−0.255 (−0.496; 0.014)	0.038	0.117	0.055 (−0.063; 0.172)	0.363	0.115	−0.082 (−0.194; 0.030)	0.153	0.120
M4	−0.382 (−0.752; −0.012)	0.043	0.182	−0.051 (−0.241; 0.139)	0.596	0.182	−0.109 (−0.229; 0.032)	0.074	0.182

M1: (crude); M2: (adjusted for M1 plus Log Total Energy); M3: (adjusted for M2 plus Body Mass Index); M4: (adjusted for M3 plus age, smoking, physical activity, alcohol intake, seasonality, education level, PC1, PC2 and PC3); M1 = model 1; M2 = model 2; M3 = model 3; M4 = model 4; PC1 = Magnesium, phosphorus and plant protein driven nutrients; PC2 = Fat and animal protein driven nutrients; PC3 = Starch, dietary fibre and B vitamin driven nutrients Fasting glucose units mmol·L^−1^ = millimoles per litre; SD = standard deviation; CI = confidence interval.

**Table 6:** Regression coefficients for glycated haemoglobin for 1 SD increase in the derived nutrient pattern scores among rural black South African women.

	Thiamine, Zinc and Plant Protein Driven Nutrients			Fat and Animal Protein Driven Nutrients			Retinol and Vitamin B12 Driven Nutrients		
	B (95% CI)	*p* Value	*R*^2^	B (95% CI)	*p* Value	*R*^2^	B (95% CI)	*p* Value	*R*^2^
M1	−0.039 (−0.117; 0.038)	0.320	0.000	0.046 (−0.031; 0.123)	0.241	0.005	0.012 (−0.065; 0.090)	0.754	0.000
M2	−0.214 (−0.384; 0.044)	0.014	0.024	0.053 (−0.031; 0.138)	0.213	0.006	0.015 (−0.065; 0.094)	0.713	0.001
M3	−0.230 (−0.392; −0.067)	0.006	0.113	0.050 (−0.030; 0.131)	0.219	0.092	0.001 (−0.075; 0.077)	0.975	0.086
M4	−0.288 (−0.543; −0.033)	0.027	0.174	−0.057 (−0.189; 0.075)	0.396	0.174	−0.018 (−0.100; 0.064)	0.662	0.174

M1: (crude); M2: (adjusted for M1 plus Log Total Energy); M3: (adjusted for M2 plus Body Mass Index); M4: (adjusted for M3 plus age, smoking, physical activity, alcohol intake, seasonality, education level, PC1, PC2 and PC3); M1 = model 1; M2 = model 2; M3 = model 3; M4 = model 4; PC1 = Magnesium, phosphorus and plant protein driven nutrients; PC2 = Fat and animal protein driven nutrients; PC3 = Starch, dietary fibre and B vitamin driven nutrients. Glycated haemoglobin unit = per cent; SD = standard deviation; CI = confidence interval.

## Supplementary Materials

The following are available online at http://www.mdpi.com/2072-6643/9/1/9/s1, Table S1. Extracted nutrient patterns and factor loadings of rural women and rural men, Table S2. Nutrient patterns and factor loadings for urban participants, Table S3. Comparison of the factors loadings in the plant driven nutrient patterns among the urban and rural participants, Table S4. Regression coefficients for fasting glucose for 1 SD increase in the derived nutrient pattern scores among urban black South African women, Table S5. Regression coefficients for glycated haemoglobin for 1 SD increase in the derived nutrient pattern scores among urban black South African women, Table S6. Regression coefficients for fasting glucose for 1 SD increase in the derived nutrient pattern scores among urban black South African men, Table S7. Regression coefficients for glycated haemoglobin for 1 SD increase in the derived nutrient pattern scores among urban black South African men.
