# Supplementary material for: Nutrient Patterns Associated with Fasting Glucose and Glycated Haemoglobin Levels in a Black South African Population
Source: Nutrients. 2017 Jan 19;9(1):9. doi: 10.3390/nu9010009 (PMC5295053; doi:10.3390/nu9010009)
Supplement: Supplementary file 1 [file nutrients-09-00009-s001.docx]

Supplementary Materials: Nutrient Patterns Associated with Fasting Glucose and Glycated Haemoglobin Levels in a Black South African Population

Tinashe Chikowore, Pedro T. Pisa, Tertia van Zyl, Edith J. M. Feskens, Edelweiss Wentzel-Viljoen and Karin R. Conradie

**Table S1.** Extracted nutrient patterns and factor loadings of rural women and rural men.

|  | **Rural Women** | | | **Rural Men** | | |
| --- | --- | --- | --- | --- | --- | --- |
| **Nutrients** | **Magnesium, Phosphorus and Plant Protein Driven Nutrients** | **Fat and Animal Protein Driven Nutrients** | **Starch, Dietary Fibre and B Vitamin Driven Nutrients** | **Thiamine, Zinc and Plant Protein Driven Nutrients** | **Fat and Animal Protein Driven Nutrients** | **Retinol and Vitamin B12 Driven Nutrients** |
| Plant protein | **0.875** | 0.211 | 0.301 | **0.924** | 0.165 | −0.030 |
| Animal protein | 0.260 | **0.831** | 0.072 | 0.153 | **0.781** | 0.371 |
| Saturated fat | 0.343 | **0.807** | −0.037 | 0.228 | **0.830** | 0.209 |
| Monounsaturated fat | 0.326 | **0.876** | 0.008 | 0.252 | **0.886** | 0.117 |
| Polyunsaturated fat | 0.301 | **0.855** | 0.099 | 0.323 | **0.830** | −0.122 |
| Cholesterol | 0.137 | **0.891** | 0.148 | −0.030 | **0.827** | 0.338 |
| Starch | 0.173 | 0.071 | **0.949** | **0.758** | −0.099 | −0.138 |
| Sugar | 0.270 | 0.352 | 0.042 | 0.164 | 0.274 | 0.218 |
| Dietary fibre | **0.680** | 0.208 | **0.608** | **0.891** | 0.157 | −0.086 |
| Calcium | **0.685** | 0.424 | 0.025 | **0.637** | 0.321 | 0.437 |
| Iron | **0.853** | 0.181 | 0.394 | **0.924** | 0.145 | −0.028 |
| Magnesium | **0.943** | 0.117 | 0.122 | **0.878** | 0.072 | 0.099 |
| Phosphorus | **0.886** | 0.334 | 0.138 | **0.850** | 0.267 | 0.204 |
| Potassium | **0.842** | 0.416 | 0.059 | **0.836** | 0.340 | 0.185 |
| Zinc | **0.855** | 0.285 | 0.337 | **0.927** | 0.261 | 0.043 |
| Retinol | −0.012 | 0.261 | −0.106 | −0.082 | 0.145 | **0.981** |
| Beta carotene | 0.080 | 0.460 | 0.059 | −0.013 | 0.311 | 0.177 |
| Thiamine | **0.775** | 0.043 | **0.546** | **0.952** | 0.028 | −0.086 |
| Riboflavin | **0.798** | 0.305 | 0.227 | **0.792** | 0.157 | 0.325 |
| Vitamin B6 | **0.520** | 0.181 | 0.756 | **0.829** | 0.108 | −0.043 |
| Folate | 0.403 | 0.135 | 0.859 | **0.781** | 0.060 | −0.080 |
| Vitamin B12 | 0.154 | **0.539** | 0.157 | −0.023 | 0.436 | **0.569** |
| Vitamin C | 0.331 | **0.605** | 0.147 | 0.254 | **0.548** | 0.065 |
| Vitamin D | 0.101 | **0.803** | 0.097 | −0.092 | **0.785** | 0.161 |
| Vitamin E | 0.190 | **0.809** | 0.193 | 0.237 | **0.769** | −0.144 |
| Explained variance % | 32.747 | 27.116 | 13.536 | 40.411 | 23.127 | 8.336 |
| Cumulative explained variance % | 32.747 | 59.863 | 73.399 | 40.411 | 63.539 | 76.180 |

Bold factor loadings used to indicate factor loadings > ± 0.47 for naming the nutrient patterns.

**Table S2.** Nutrient patterns and factor loadings for urban participants.

|  | Urban Women | | | Urban Man | | |
| --- | --- | --- | --- | --- | --- | --- |
| Nutrients | **Thiamine, Starch and Folate Driven Nutrients** | **Fat and Animal Protein Driven Nutrients** | **Retinol, Vitamin B12 and Calcium Driven Nutrients** | **Thiamine, Zinc and Plant Protein Driven Nutrients** | **Fat and Animal Protein Driven Nutrients** | **Beta Carotene and Vitamin C Driven Nutrients** |
| Plant protein | **0.877** | 0.288 | 0.132 | **0.896** | 0.272 | 0.135 |
| Animal protein | 0.347 | **0.795** | 0.224 | 0.408 | **0.737** | 0.211 |
| Saturated fat | 0.363 | **0.746** | 0.290 | 0.412 | **0.741** | 0.251 |
| Monounsaturated fat | 0.366 | **0.776** | 0.185 | 0.408 | **0.766** | 0.282 |
| Polyunsaturated fat | 0.444 | **0.720** | 0.129 | 0.375 | **0.789** | 0.240 |
| Cholesterol | 0.261 | **0.827** | 0.252 | 0.217 | **0.831** | 0.103 |
| Starch | **0.898** | 0.057 | 0.016 | **0.898** | 0.108 | 0.095 |
| Sugar | 0.439 | 0.378 | 0.255 | 0.365 | 0.423 | 0.465 |
| Dietary fibre | **0.853** | 0.278 | 0.125 | **0.879** | 0.267 | 0.208 |
| Calcium | 0.405 | 0.434 | **0.512** | 0.435 | 0.449 | 0.142 |
| Iron | **0.850** | 0.381 | 0.169 | **0.903** | 0.313 | 0.182 |
| Magnesium | **0.841** | 0.320 | 0.164 | **0.863** | 0.240 | 0.186 |
| Phosphorus | **0.709** | **0.520** | 0.291 | **0.759** | 0.452 | 0.214 |
| Potassium | **0.684** | **0.498** | 0.267 | **0.736** | 0.446 | 0.327 |
| Zinc | **0.815** | **0.466** | 0.150 | **0.859** | 0.395 | 0.213 |
| Retinol | 0.077 | 0.270 | **0.950** | 0.053 | 0.272 | 0.072 |
| Beta carotene | 0.298 | 0.388 | 0.124 | 0.312 | 0.297 | **0.885** |
| Thiamine | **0.914** | 0.276 | 0.114 | **0.942** | 0.222 | 0.165 |
| Riboflavin | **0.612** | **0.520** | 0.371 | **0.744** | 0.409 | 0.197 |
| Vitamin B6 | **0.833** | 0.366 | 0.160 | **0.861** | 0.301 | 0.270 |
| Folate | **0.889** | 0.257 | 0.151 | **0.889** | 0.298 | 0.122 |
| Vitamin B12 | 0.256 | **0.558** | **0.611** | 0.352 | **0.549** | 0.141 |
| Vitamin C | 0.435 | 0.392 | 0.163 | 0.401 | 0.408 | **0.517** |
| Vitamin D | 0.150 | **0.763** | 0.213 | 0.129 | **0.804** | 0.054 |
| Vitamin E | 0.341 | **0.743** | 0.077 | 0.275 | **0.823** | 0.221 |
| Explained variance % | 38.984 | 27.351 | 9.426 | 41.885 | 26.247 | 8.370 |
| Cumulative explained variance % | 38.984 | 66.335 | 75.760 | 41.885 | 68.132 | 76.502 |

Bold factor loadings used to indicate factor loadings >±0.47 for naming the nutrient patterns.

**Table S3.** Comparison of the factors loadings in the plant driven nutrient patterns among the urban and rural participants.

|  | **Rural Men** | **Urban Men** | **Urban Females** | **Rural Women** | |
| --- | --- | --- | --- | --- | --- |
| **Nutrients** | **Thiamine, Zinc and Plant Protein Driven Nutrients** | **Thiamine, Zinc and Plant Protein Driven Nutrients** | **Thiamine, Starch and Folate Driven Nutrients** | **Magnesium, Phosphorus and Plant Protein Driven Nutrients** | **Starch, Dietary Fibre and B Vitamin Driven Nutrients** |
| Plant protein | 0.924 | 0.896 | 0.877 | 0.875 | 0.301 |
| Animal protein | 0.153 | **0.408** | **0.347** | **0.260** | 0.072 |
| Saturated fat | 0.228 | **0.412** | **0.363** | **0.343** | **−0.037** |
| Monounsaturated fat | 0.252 | **0.408** | **0.366** | **0.326** | 0.008 |
| Polyunsaturated fat | 0.323 | 0.375 | 0.444 | 0.301 | 0.099 |
| Cholesterol | **−0.030** | 0.217 | 0.261 | 0.137 | 0.148 |
| Starch | 0.758 | 0.898 | 0.898 | 0.173 | 0.949 |
| Sugar | 0.164 | **0.365** | **0.439** | **0.270** | 0.042 |
| Dietary fibre | 0.891 | 0.879 | 0.853 | 0.680 | 0.608 |
| Calcium | 0.637 | 0.435 | 0.405 | 0.685 | 0.025 |
| Iron | 0.924 | 0.903 | 0.850 | 0.853 | 0.394 |
| Magnesium | 0.878 | 0.863 | 0.841 | 0.943 | 0.122 |
| Phosphorus | 0.850 | 0.759 | 0.709 | 0.886 | 0.138 |
| Potassium | 0.836 | 0.736 | 0.684 | 0.842 | 0.059 |
| Zinc | 0.927 | 0.859 | 0.815 | 0.855 | 0.337 |
| Retinol | −0.082 | 0.053 | 0.077 | −0.012 | −0.106 |
| Beta carotene | −0.013 | 0.312 | 0.298 | 0.080 | 0.059 |
| Thiamine | 0.952 | 0.942 | 0.914 | 0.775 | 0.546 |
| Riboflavin | 0.792 | 0.744 | 0.612 | 0.798 | 0.227 |
| Vitamin B6 | 0.829 | 0.861 | 0.833 | 0.520 | 0.756 |
| Folate | 0.781 | 0.889 | 0.889 | 0.403 | 0.859 |
| Vitamin B12 | −0.023 | 0.352 | 0.256 | 0.154 | 0.157 |
| Vitamin C | 0.254 | 0.401 | 0.435 | 0.331 | 0.147 |
| Vitamin D | −0.092 | 0.129 | 0.150 | 0.101 | 0.097 |
| Vitamin E | 0.237 | 0.275 | 0.341 | 0.190 | 0.193 |
| Explained variance % | **40.411** | **41.885** | **38.984** | **32.747** | **13.536** |

Bold factor loadings used for comparing the nutrient patterns.

**Table S4.** Regression coefficients for fasting glucose for 1 SD increase in the derived nutrient pattern scores among urban black South African women.

|  | **Thiamine, Starch and Folate Driven Nutrients** | | | **Fat and Animal Protein Driven Nutrients** | | | **Retinol, Vitamin B12 and Calcium Driven Nutrients** | | |
| --- | --- | --- | --- | --- | --- | --- | --- | --- | --- |
|  | **B (95% CI)** | ***p* Value** | ***R*^2^** | **B (95% CI)** | ***p* Value** | ***R*^2^** | **B (95% CI)** | ***p* Value** | ***R*^2^** |
| **M1** | −0.198 (−0.372; −0.024) | 0.025 | 0.011 | 0.072 (−0.247; 0.104) | 0.423 | 0.000 | −0.074 (−0.246; 0.097) | 0.395 | 0.002 |
| **M2** | −0.139 (−0.414; 0.136) | 0.321 | 0.013 | 0.048 (−0.160; 0.256) | 0.649 | 0.003 | −0.047 (−0.225; 0.131) | 0.607 | 0.011 |
| **M3** | −0.084 (−0.358; 0.190) | 0.547 | 0.037 | 0.019 (−0.187; 0.225) | 0.859 | 0.037 | −0.039 (−0.215; 0.137) | 0.660 | 0.037 |
| **M4** | −0.227 (−0.683; 0.230) | 0.329 | 0.062 | −0.058 (−0.390; 0.274) | 0.732 | 0.062 | −0.058 (−0.270; 0.154) | 0.591 | 0.062 |

M1: (crude); M2: (adjusted for M1 plus Log Total Energy); M3: (Adjusted for M2 plus Body Mass Index); M4: (Adjusted for M3 plus age, smoking, physical activity, alcohol intake, seasonality, education level, PC1, PC2 and PC3); M1 = model 1; M2 = model 2; M3 = model 3; M4 = model 4; PC1 = Magnesium, phosphorus and plant protein driven nutrients; PC2 = Fat and animal protein driven nutrients; PC3 = Starch, dietary fibre and B vitamin driven nutrients Fasting glucose units mmol·L^−1^ = millimoles per litre; SD = standard deviation; CI = confidence interval.

**Table S5.** Regression coefficients for glycated haemoglobin for 1 SD increase in the derived nutrient pattern scores among urban black South African women.

|  | **Thiamine, Starch and Folate Driven Nutrients** | | | **Fat and Animal Protein Driven Nutrients** | | | **Retinol, Vitamin B12 and Calcium Driven Nutrients** | | |
| --- | --- | --- | --- | --- | --- | --- | --- | --- | --- |
|  | **B (95% CI)** | ***p* Value** | ***R*^2^** | **B (95% CI)** | ***p* Value** | ***R*^2^** | **B (95% CI)** | ***p* Value** | ***R*^2^** |
| **M1** | −0.060 (−0.160; 0.041) | 0.246 | 0.003 | −0.033 (−0.134; 0.068) | 0.521 | 0.001 | −0.035 (−0.135; 0.066) | 0.500 | 0.001 |
| **M2** | −0.033 (−0.192; 0.126) | 0.679 | 0.003 | −0.001 (−0.121; 0.119) | 0.813 | 0.003 | −0.020 (−0.125; 0.085) | 0.703 | 0.003 |
| **M3** | 0.017 (−0.139; 0.173) | 0.833 | 0.054 | −0.028 (−0.146; 0.089) | 0.635 | 0.037 | −0.018 (−0.120; 0.085) | 0.733 | 0.054 |
| **M4** | −0.001 (−0.264; 0.263) | 0.994 | 0.106 | −0.018 (−0.209; 0.174) | 0.854 | 0.106 | −0.015 (−0.139; 0.109) | 0.807 | 0.106 |

M1: (crude); M2: (adjusted for M1 plus Log Total Energy); M3: (Adjusted for M2 plus Body Mass Index); M4: (Adjusted for M3 plus age, smoking, physical activity, alcohol intake, seasonality, education level, PC1, PC2 and PC3); M1 = model 1; M2 = model 2; M3 = model 3; M4 = model 4; PC1 = Magnesium, phosphorus and plant protein driven nutrients; PC2 = Fat and animal protein driven nutrients; PC3 = Starch, dietary fibre and B vitamin driven nutrients Glycated haemoglobin unit = %; SD = standard deviation; CI = confidence interval.

**Table S6.** Regression coefficients for fasting glucose for 1 SD increase in the derived nutrient pattern scores among urban black South African men.

|  | **Thiamine, Zinc and Plant Protein Driven Nutrients** | | | **Fat and Animal Protein Driven Nutrients** | | | **Beta Carotene and Vitamin C Driven Nutrients** | | |
| --- | --- | --- | --- | --- | --- | --- | --- | --- | --- |
|  | **B (95% CI)** | ***p* Value** | ***R*^2^** | **B (95% CI)** | ***p* Value** | ***R*^2^** | **B (95% CI)** | ***p* Value** | ***R*^2^** |
| **M1** | −0.123 (−0.345; 0.100) | 0.279 | 0.004 | 0.039 (−0.187; 0.264) | 0.737 | 0.000 | 0.018 (−0.207; 0.244) | 0.873 | 0.000 |
| **M2** | −0.159 (−0.541; 0.222) | 0.412 | 0.004 | 0.098 (−0.157; 0.353) | 0.449 | 0.004 | 0.044 (−0.193; 0.281) | 0.714 | 0.002 |
| **M3** | −0.069 (−0.453; 0.316) | 0.726 | 0.025 | 0.057 (−0.198; 0.311) | 0.662 | 0.025 | 0.018 (−0.218; 0.253) | 0.883 | 0.025 |
| **M4** | −0.080 (−0.844; 0.685) | 0.838 | 0.087 | 0.105 (−0.368; 0.578) | 0.662 | 0.087 | −0.096 (−0.438; 0.246) | 0.582 | 0.087 |

M1: (crude); M2: (adjusted for M1 plus Log Total Energy); M3: (Adjusted for M2 plus Body Mass Index); M4: (Adjusted for M3 plus age, smoking, physical activity, alcohol intake, seasonality, education level, PC1, PC2 and PC3); M1 = model 1; M2 = model 2; M3 = model 3; M4 = model 4; PC1 = Magnesium, phosphorus and plant protein driven nutrients; PC2 = Fat and animal protein driven nutrients; PC3 = Starch, dietary fibre and B vitamin driven nutrients Fasting glucose units mmol·L^−1^ = millimoles per litre; SD = standard deviation; CI = confidence interval.

**Table S7.** Regression coefficients for glycated haemoglobin for 1 SD increase in the derived nutrient pattern scores among urban black South African men.

|  | **Thiamine, Zinc and Plant Protein Driven Nutrients** | | | **Fat and Animal Protein Driven Nutrients** | | | **Beta Carotene and Vitamin C Driven Nutrients** | | |
| --- | --- | --- | --- | --- | --- | --- | --- | --- | --- |
|  | **B (95% CI)** | ***p* Value** | ***R*^2^** | **B (95% CI)** | ***p* Value** | ***R*^2^** | **B (95% CI)** | ***p* Value** | ***R*^2^** |
| **M1** | 0.045 (−0.041; 0.131) | 0.303 | 0.003 | −0.001 (−0.088; 0.085) | 0.973 | 0.000 | −0.003 (−0.089; 0.083) | 0.937 | 0.000 |
| **M2** | 0.077 (−0.070; 0.224) | 0.301 | 0.005 | −0.018 (−0.116; 0.081) | 0.724 | 0.002 | −0.012 (−0.102; 0.078) | 0.789 | 0.001 |
| **M3** | 0.119 (−0.029; 0.266) | 0.166 | 0.030 | −0.035 (−0.133; 0.063) | 0.483 | 0.024 | −0.022 (−0.112; 0.067) | 0.622 | 0.024 |
| **M4** | 0.179 (−0.116; 0.473) | 0.233 | 0.094 | 0.054 (−0.128; 0.235) | 0.559 | 0.094 | −0.005 (−0.136; 0.126) | 0.938 | 0.094 |

M1: (crude); M2: (adjusted for M1 plus Log Total Energy); M3: (Adjusted for M2 plus Body Mass Index); M4: (Adjusted for M3 plus age, smoking, physical activity, alcohol intake, seasonality, education level, PC1, PC2 and PC3); M1 = model 1; M2 = model 2; M3 = model 3; M4 = model 4; PC1 = Magnesium, phosphorus and plant protein driven nutrients; PC2 = Fat and animal protein driven nutrients; PC3 = Starch, dietary fibre and B vitamin driven nutrients Glycated haemoglobin unit = %; SD = standard deviation; CI = confidence interval.
